# Tuning the properties of hydrogen-bonded block copolymer worm gels prepared *via* polymerization-induced self-assembly†

**DOI:** 10.1039/d1sc03156b

**Published:** 2021-08-05

**Authors:** Eleanor Raphael, Matthew J. Derry, Michael Hippler, Steven P. Armes

**Affiliations:** Chemistry Department, University of Sheffield Dainton Building, Brook Hill Sheffield South Yorkshire S3 7HF UK m.derry@aston.ac.uk s.p.armes@sheffield.ac.uk

## Abstract

Polymerization-induced self-assembly (PISA) is exploited to design hydrogen-bonded poly(stearyl methacrylate)-poly(benzyl methacrylate) [PSMA-PBzMA] worm gels in *n*-dodecane. Using a carboxylic acid-based RAFT agent facilitates hydrogen bonding between neighboring worms to produce much stronger physical gels than those prepared using the analogous methyl ester-based RAFT agent. Moreover, tuning the proportion of these two types of end-groups on the PSMA chains enables the storage modulus (*G*′) of a 20% w/w worm gel to be tuned from ∼4.5 kPa up to ∼114 kPa. This is achieved *via* two complementary routes: (i) an *in situ* approach using binary mixtures of acid- and ester-capped PSMA stabilizer chains during PISA or (ii) a post-polymerization processing strategy using a thermally-induced worm-to-sphere transition to mix acid- and ester-functionalized spheres at 110 °C that fuse to form worms on cooling to 20 °C. SAXS and rheology studies of these hydrogen-bonded worm gels provide detailed insights into their inter-worm interactions and physical behavior, respectively. In the case of the carboxylic acid-functionalized worms, SAXS provides direct evidence for additional inter-worm interactions, while rheological studies confirm both a significant reduction in critical gelation concentration (from approximately 10% w/w to 2–3% w/w) and a substantial increase in critical gelation temperature (from 41 °C to 92 °C). It is remarkable that a rather subtle change in the chemical structure results in such improvements in gel strength, gelation efficiency and gel cohesion.

## Abstract 2

Carboxylic acid-capped diblock copolymer worms are prepared in *n*-dodecane *via* polymerization-induced self-assembly. Varying the proportion of terminal carboxylic acid groups modulates the inter-worm H-bonding interactions and hence the gel modulus.

## Graphical abstract

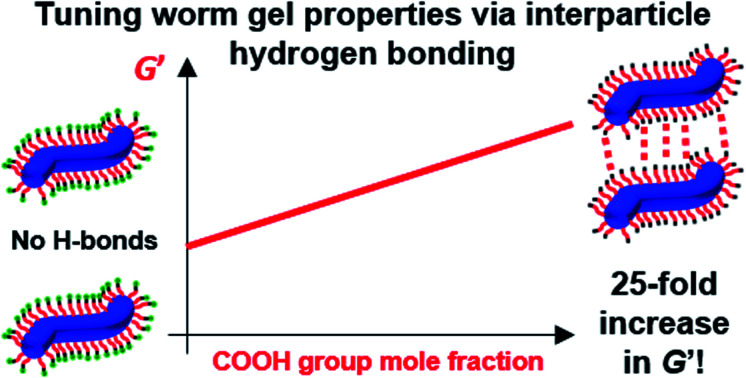

## Introduction

Hydrogen bonding is widely regarded as the most important example of a non-covalent interaction between molecules.^1–4^ Indeed, hydrogen bonding is an essential component of the secondary structure of DNA *via* formation of complementary base pairs.^5^ This well-known phenomenon has been exploited in polymer science by various research groups to generate supramolecular morphologies, as well as to tune mechanical properties.^6–10^ For example, 2-ureido-4[1*H*]-pyrimidone (UPy) motifs have been used to introduce highly cooperative hydrogen bonding interactions in either aqueous or non-aqueous media.^6,9,11–14^ Thus, Meijer and co-workers^9^ prepared poly(ethylene glycol)-based hydrogels by introducing UPy groups into amphiphilic copolymers in order to form strong hydrogen bonds that act as physical cross-links. Leibler's group^7,8^ reported the design of hydrogen-bonded synthetic rubbers using renewable starting materials that exhibit both self-healing and thermoreversible behavior. Kornfield and co-workers^15^ utilized charge-assisted hydrogen bonding to design complementary low molecular weight telechelic precursors that self-assemble to form high molecular weight ‘megasupramolecules’ in non-polar media. This approach confers a significant thickening effect, while irreversible shear-induced degradation can be avoided *via* a ‘self-healing’ mechanism. Hence these hydrogen-bonded nanostructures significantly out-perform high molecular weight polyisobutylene additives as anti-misting agents for jet fuel. Similar hydrogen bonding interactions between imidazole and carboxylic acid groups have been exploited for the formation of hydrogels with enhanced mechanical properties,^16^ while Ikkala and co-workers^17,18^ reported exquisite control over multiple length scales *via* hydrogen bonding-mediated self-assembly of a 1 : 1 methanesulfonic acid/poly(4-vinylpyridine) using varying amounts of pentadecylphenol. The discovery of living anionic polymerization,^19,20^ and more recently the development of reversible-deactivation radical polymerization techniques,^21,22^ has enabled the design of a remarkably wide range of well-defined functional block copolymers, which exhibit spontaneous self-assembly either in the bulk^23,24^ and/or in solution.^25,26^ For example, the formation of diblock copolymer spheres in solvents that are selective for one of the two blocks has been known for more than fifty years.^27^ In 1999, Bates and co-workers^28^ reported that aqueous dispersions of highly anisotropic poly(ethylene oxide)-polybutadiene worms formed free-standing viscoelastic gels above a certain critical copolymer concentration. Since this seminal study, block copolymer worms have been evaluated for drug delivery,^29,30^ as sterilizable hydrogels^31^ for 3D cell culture^32^ and stem cell storage,^33^ for cryopreservation of red blood cells,^34^ as superflocculants for micrometer-sized particles,^35^ for viscosity modification,^36^ for reinforcement of latex films^37^ and as model Pickering emulsifiers.^38,39^ This sub-field has been reviewed by Davis and co-workers^40^ and more recently by Tian *et al.*^41^

Traditionally, block copolymer self-assembly in solution has been achieved by post-polymerization processing techniques such as a solvent switch or thin film rehydration, which usually only enable the preparation of rather dilute copolymer dispersions.^25,42,43^ In contrast, polymerization-induced self-assembly (PISA) enables the rational synthesis of block copolymer nano-objects at relatively high copolymer concentrations (up to 50% w/w).^44–47^ In particular, reversible addition–fragmentation chain transfer (RAFT) polymerization^48,49^ has enabled the efficient PISA synthesis of a wide range of functional block copolymer spheres, worms or vesicles in aqueous,^50–53^ alcoholic^54–59^ or non-polar^60–64^ media. Typically, the worm morphology occupies rather narrow phase space.^60–63^ However, Rieger and co-workers recently designed a functional RAFT agent in order to place bis-urea ‘stickers’ within the core-forming block; introducing this hydrogen bonding motif enables the worm phase space to be significantly expanded for an aqueous PISA formulation.^65^ Alternatively, constructing an appropriate pseudo-phase diagram based on PISA syntheses provides a reliable means of targeting the otherwise elusive worm morphology.^59–63^ This systematic approach has led directly to many more examples of well-defined block copolymer worms being reported.^66–70^ An additional method of controlling nanoparticle morphology during PISA involves the judicious selection of chain-end functionality.^71^ It is now recognized that many PISA formulations based on RAFT dispersion polymerization afford thermoresponsive diblock copolymer worms^72^ in aqueous,^31,73^ alcoholic,^74^ or non-polar^67,68^ media. In particular, heating a dispersion of poly(lauryl methacrylate)-poly(benzyl methacrylate) worms in *n*-dodecane induces a worm-to-sphere morphology transition, which can be fully reversible if conducted at sufficiently high copolymer concentration.^67^

**Scheme 1:**
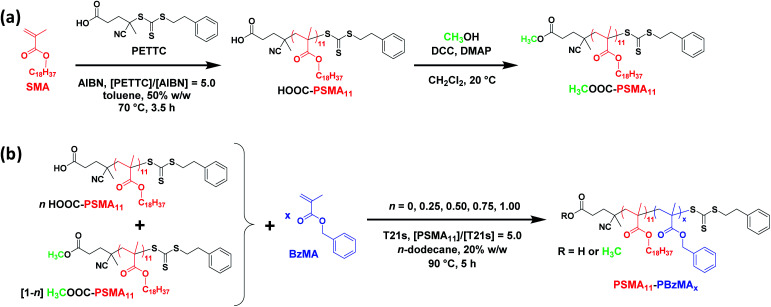
(a) Synthesis of HOOC-PSMA_11_ macro-CTA *via* RAFT solution polymerization of SMA in toluene at 70 °C and the subsequent preparation of its methyl ester analogue, H_3_COOC-PSMA_11_ macro-CTA, *via* esterification. (b) Synthesis of a series of PSMA_11_-PBzMA_*x*_ diblock copolymer nano-objects *via* RAFT dispersion polymerization of BzMA in *n*-dodecane at 90 °C using various binary mixtures of HOOC-PSMA_11_ and H_3_COOC-PSMA_11_ macro-CTAs.

**Scheme 2:**
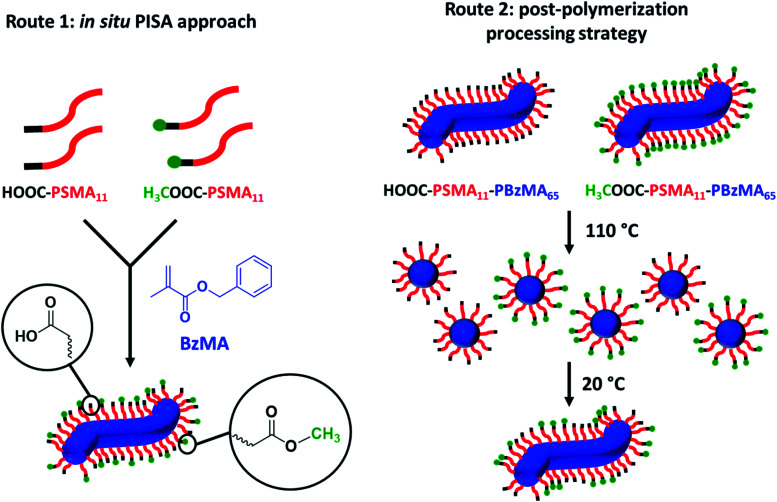
Schematic representation of the two synthetic routes used herein to prepare the two series of PSMA-PBzMA worms containing varying proportions of carboxylic acid end-groups. Both routes are based on the principle of entropic mixing. Route 1 utilizes a binary mixture of HOOC-PSMA_11_ and H_3_COOC-PSMA_11_ precursors during the RAFT dispersion polymerization of BzMA; this approach results in a statistical distribution of carboxylic acid end-groups located at the outer surface of each sterically-stabilized worm. Route 2 involves heating two ‘masterbatch’ 20% w/w dispersions comprising HOOC-PSMA_11_-PBzMA_65_ and H_3_COOC-PSMA_11_-PBzMA_65_ worm gels up to 110 °C to induce a worm-to-sphere transition (and concomitant degelation) in each case. These two free-flowing fluids of spherical nanoparticles were then mixed together in various proportions at this temperature to produce the desired range of carboxylic acid/methyl ester molar ratios. On cooling to 20 °C, a sphere-to-worm transition occurs *via* 1D stochastic fusion of multiple (mixed) spheres to produce ‘hybrid’ segmented worms comprising spatially-localized patches of steric stabilizer chains bearing carboxylic acid end-groups.

The present work focuses on diblock copolymer worms that form free-standing gels at sufficiently high copolymer concentration owing to a percolating network arising from multiple inter-worm contacts.^75^ Hydrogen bonding is particularly strong in non-polar media,^76^ with one well-known example of such a non-covalent interaction being the dimerization of acetic acid in benzene.^77^ In principle, the synthesis of diblock copolymer worms *via* RAFT-mediated PISA in non-polar media provides an opportunity to form stronger gels by introducing appropriate inter-worm attractive forces. In this context, it is noteworthy that selecting an appropriate RAFT chain transfer agent (CTA) enables carboxylic acid groups to be conveniently introduced at the end of every steric stabilizer chain (see Scheme 1). For methacrylates, this RAFT CTA can typically be either a dithioester or trithiocarbonate. For the purposes of the present work, a carboxylic acid-functional trithiocarbonate, 4-cyano-4-(2-phenylethanesulfanylthiocarbonyl)sulfanylpentanoic acid (PETTC), was utilized. Thus, when the copolymer concentration exceeds the critical gelation concentration, this should lead to the formation of carboxylic acid dimers at the point where neighboring worms just touch each other to form the 3D percolating gel network.^75^ Moreover, synthesis of the equivalent diblock copolymer worms using a methyl ester-based RAFT CTA offers a suitable reference gel for which no inter-worm hydrogen bonding interactions are possible. Thus, systematic variation of the carboxylic acid/methyl ester molar ratio should enable modulation of the inter-worm interactions and hence provide fine control over the physical properties of the worm gels. Alternatively, the thermoresponsive nature of such diblock copolymer worms can be exploited to adjust the worm gel strength. In principle, this is achieved by heating concentrated dispersions of carboxylic acid- and methyl ester-functionalized worms up to 110 °C in turn to induce a worm-to-sphere transition in each case, followed by mixing the resulting hot free-flowing fluids together in varying proportions. Subsequent cooling leads to the formation of a series of ‘hybrid’ segmented worm gels *via* stochastic 1D fusion of the carboxylic acid- and methyl ester-functionalized spheres (see Scheme 2). This complementary approach should also enable the worm gel strength to be tuned over a wide range. Herein we examine these new concepts by performing rheological measurements on well-characterized diblock copolymer worm dispersions in a model high boiling point solvent, *n*-dodecane.

## Results and discussion

### Synthesis of HOOC-PSMA_11_ and H_3_COOC-PSMA_11_ homopolymer precursors *via* RAFT solution polymerization of SMA

A kinetic study of the RAFT solution polymerization of SMA in toluene at 70 °C was conducted by periodically removing aliquots of the reaction mixture for analysis over a 7 h period (see Fig. S1†). Monomer conversions were calculated using ^1^H NMR spectroscopy by comparing the integrated monomer vinyl proton signals at 5.6 and 6.2 ppm with the two oxymethylene protons assigned to the monomer/polymer at 4.0 ppm. A monotonic increase in monomer conversion with polymerization time was observed (see Fig. S1a†). There are two distinct regimes: initial relatively slow polymerization for the first 100 min, followed by a faster rate of polymerization that obeys first-order kinetics with respect to SMA, as judged by the linear semi-logarithmic plot. The GPC curves shown in Fig. S1b† were analyzed using a refractive index detector and poly(methyl methacrylate) calibration standards: a linear evolution in *M*_*n*_ with conversion was observed (see Fig. S1c†) and a relatively narrow molecular weight distribution was obtained for the final PSMA homopolymer (*M*_*n*_ = 3100 g mol^−1^; *M*_w_/*M*_*n*_ = 1.18; 93% conversion). Informed by this kinetic study, SMA was polymerized on a 40 gram scale under the same conditions and quenched after 6 h (76% conversion) to ensure retention of the trithiocarbonate-based RAFT chain-ends. After purification *via* precipitation into excess ethanol, the resulting HOOC-PSMA macro-CTA had a mean degree of polymerization (DP) of 11 as judged by ^1^H NMR spectroscopy, while THF GPC analysis indicated an *M*_*n*_ of 5400 g mol^−1^ and an *M*_w_/*M*_*n*_ of 1.11.§§This *M*_*n*_ value is somewhat larger than that obtained at 93% monomer conversion during the kinetic study (see Fig. S1c). This is because a purification *via* precipitation into excess ethanol removes lower molecular weight PSMA oligomers, which remain soluble in this solvent. Esterification of this HOOC-PSMA_11_ precursor using excess methanol produced the corresponding H_3_COOC-PSMA_11_ macro-CTA (see Scheme 1a). ^1^H NMR spectroscopy confirmed successful end-group derivatization: comparison of the integrated terminal methyl ester proton signal at 3.7 ppm to that of the oxymethylene proton signal assigned to the SMA repeat units at 4.0 ppm indicated that the degree of esterification of the carboxylic acid end-groups was 97% (see Fig. S2†). Importantly, this synthetic strategy produces two chemically identical steric stabilizer blocks that differ only in the nature of their end-groups. Indeed, the THF GPC curves recorded for these two precursors overlay almost precisely (see Fig. S3†).

### Synthesis of HOOC-PSMA_11_-PBzMA_*x*_ and H_3_COOC-PSMA_11_-PBzMA_*x*_ diblock copolymer nano-objects

**Fig. 1:**
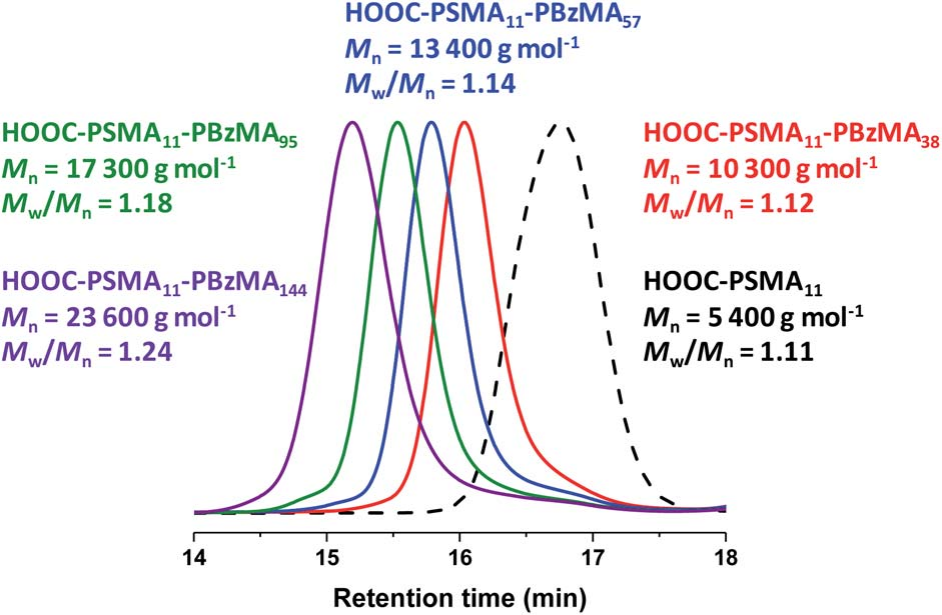
THF GPC curves recorded for selected HOOC-PSMA_11_-PBzMA_*x*_ diblock copolymers prepared *via* RAFT dispersion polymerization of BzMA in *n*-dodecane at 90 °C using the HOOC-PSMA_11_ precursor. Conditions: 20% w/w solids, [HOOC-PSMA_11_]/[T21s] molar ratio = 5.0, reaction time = 5 h. The GPC curve recorded for the HOOC-PSMA_11_ homopolymer precursor (black dashed curve) is also shown as a reference.

A series of PSMA_11_-PBzMA_*x*_ diblock copolymer nano-objects were synthesized *via* RAFT dispersion polymerization of BzMA using the HOOC-PSMA_11_ precursor at 90 °C in *n*-dodecane (see Scheme 1b, where *n* = 1.00). In all cases, the final BzMA conversion was at least 95% and THF GPC analysis indicated that narrow molecular weight distributions (*M*_w_/*M*_*n*_ < 1.25) were obtained when targeting PBzMA DPs up to 150 (see Fig. 1). Depending on the target PBzMA DP, this series of HOOC-PSMA_11_-PBzMA_*x*_ diblock copolymers self-assembled *in situ* to form either spherical (*x* ≤ 38), worm-like (52 ≤ *x* ≤ 65) or vesicular (*x* ≥ 95) morphologies (see Fig. S4†). These observations are in generally good agreement with those reported for a series of closely-related PSMA_13_-PBzMA_*x*_ diblock copolymer nano-objects prepared in mineral oil using a dithiobenzoate-based RAFT agent.^63^ Importantly, the corresponding series of H_3_COOC-PSMA_11_-PBzMA_*x*_ diblock copolymers prepared using the same PISA protocol also formed pure worms for 53 ≤ *x* ≤ 65 (see Fig. S5†). Both the carboxylic acid and methyl ester end-groups are located on the outer surface of these sterically-stabilized worms (see Scheme 2). Thus, this offers an unprecedented opportunity to modulate the physical properties of the free-standing worm gels that are formed as a result of multiple inter-worm contacts.^75^ More specifically, conducting a series of PISA syntheses utilizing a binary mixture of HOOC-PSMA_11_ and H_3_COOC-PSMA_11_ macro-CTAs enables systematic variation of the carboxylic acid/methyl ester molar ratio. In principle, this should enable fine-tuning of the inter-worm interactions, which in turn influence the physical properties of the worm gels.

### Tuning the properties of hydrogen-bonded PSMA-PBzMA worm gels

Firstly, a series of PSMA_11_-PBzMA_65_ worms comprising varying amounts of carboxylic acid and methyl ester end-groups were prepared *in situ via* PISA by utilizing a binary mixture of the HOOC-PSMA_11_ and H_3_COOC-PSMA_11_ precursors (see Route 1 in Scheme 2). Systematic variation of the HOOC-PSMA_11_/H_3_COOC-PSMA_11_ molar ratio enabled the proportion of carboxylic acid end-groups to be fine-tuned from 0 to 100 mol% for a series of five worm gels. Alternatively, a series of five PSMA_11_-PBzMA_65_ worm gels comprising steric stabilizer chains bearing carboxylic acid end-groups were prepared *via* a post-polymerization processing strategy whereby two separate ‘masterbatches’ comprising 20% w/w dispersions of HOOC-PSMA_11_-PBzMA_65_ and H_3_COOC-PSMA_11_-PBzMA_65_ worm gels were heated to 110 °C (see Route 2 in Scheme 2). In both cases, this thermal treatment induced a worm-to-sphere transition with concomitant *in situ* degelation.^67^ The resulting two free-flowing fluids were subsequently mixed together in various proportions at this temperature and then each dispersion was allowed to cool to ambient temperature to induce a sphere-to-worm transition, with the resulting copolymer dispersion forming a free-standing gel at 20 °C. Assuming that entropic mixing occurs (rather than self-sorting), the stochastic 1D fusion of multiple (mixed) spheres is expected to produce a series of ‘hybrid’ segmented worms comprising spatially-localized patches of carboxylic acid end-groups.

**Fig. 2:**
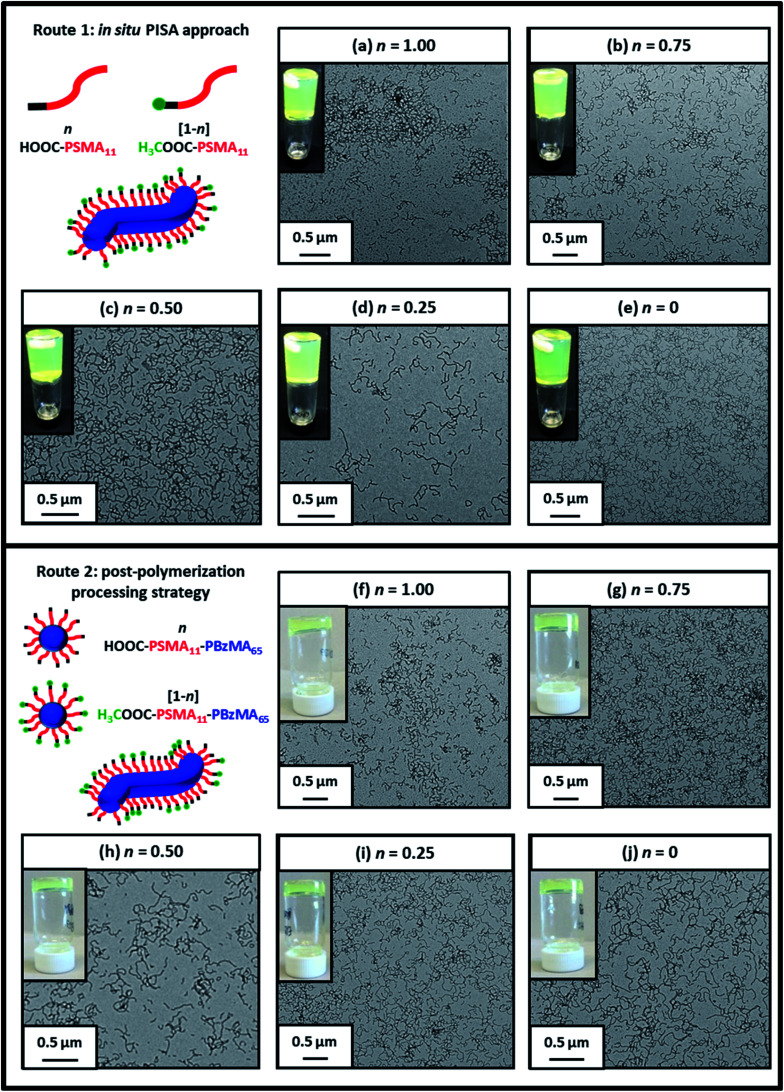
Representative transmission electron micrographs recorded for 0.10% w/w dispersions of the ten examples of PSMA-PBzMA diblock copolymer worms prepared using Route 1 and Route 2 in this study (see Scheme 2). Insets show digital photographs recorded for the corresponding ten worm gels at 10% w/w solids. This so-called tube inversion test confirms their free-standing nature at ambient temperature.

**Fig. 3:**
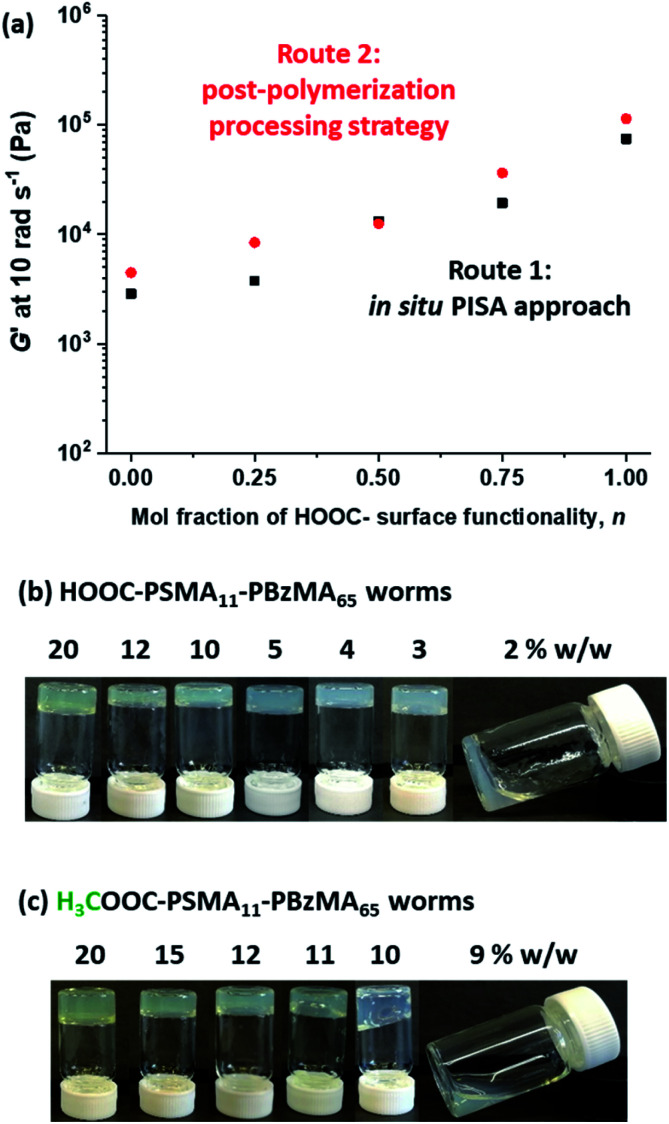
(a) Effect of varying the mole fraction of carboxylic acid end-groups on the storage modulus, *G*′, for 20% w/w PSMA_11_-PBzMA_65_ worm gels prepared *via* Routes 1 and 2 [*G*′ data recorded at an angular frequency of 10 rad s^−1^]. Digital images recorded for HOOC-PSMA_11_-PBzMA_65_ (b) and H_3_COOC-PSMA_11_-PBzMA_65_ (c) worms dispersed in *n*-dodecane at various copolymer concentrations.

Five worm gels were prepared using Route 1 and Route 2, respectively. In each case, the mole fraction of carboxylic acid end-groups (*n*) was adjusted to be 0, 0.25, 0.50, 0.75 and 1.00. Importantly, transmission electron microscopy (TEM) studies confirmed that a pure worm morphology was obtained in each case (see Fig. 2). Moreover, each of these ten copolymer dispersions formed relatively transparent free-standing worm gels at 20 °C (see Fig. 2, inset images). Each of the 20% w/w copolymer dispersions prepared by either Route 1 or Route 2 exhibit a pure worm morphology and formed free-standing gels at ambient temperature. The physical properties of these worm gels were assessed *via* oscillatory rheology studies. Angular frequency sweeps from 0.1 to 100 rad s^−1^ were performed at 25 °C using a fixed strain amplitude of 1.0% (see Fig. S6†). Importantly, the thermal history of these worm gels was removed by heating to 110 °C, then allowing to cool to 25 °C over a 24 h period before conducting the angular frequency sweeps.^73^ The storage modulus (*G*′) remained significantly greater than the loss modulus (*G*′′) over the entire range of angular frequencies, thus indicating that each dispersion was a true gel. Furthermore, these worm gels exhibit linear viscoelasticity in this region, as judged by the relatively weak angular frequency dependence for *G*′. For both series of worm gels, substantially higher *G*′ values were obtained when increasing the carboxylic acid end-group content (see Fig. 3a). Thus, a 20% w/w worm gel comprising solely HOOC-PSMA_11_-PBzMA_65_ exhibited a *G*′ of ∼114 kPa, whereas that for a 20% w/w worm gel comprising solely H_3_COOC-PSMA_11_-PBzMA_65_ was ∼4.5 kPa. This approximate 25-fold increase in *G*′ suggests significantly greater inter-worm interactions for the former gel. Interestingly, the worm gels prepared *via* Route 2 typically exhibit higher *G*′ values than the equivalent worm gels prepared *via* Route 1. In principle, this suggests that a relatively high local concentration of carboxylic acid end-groups favors the formation of multiple carboxylic acid dimers at the point(s) of contact between neighboring worms. However, in principle the *G*′ values obtained for the two pairs of worm gels containing no carboxylic acid groups (*n* = 0) and solely carboxylic acid groups (*n* = 1.00) should be identical. Thus the observed difference between each pair of measurements shown in Fig. 3a most likely indicates the experimental uncertainty in these rheological experiments (estimated to be 30–50%). In this context, it is worth emphasizing that the 25-fold boost in *G*′ is much larger than this experimental uncertainty.

Temperature-dependent oscillatory rheology studies provide further evidence for hydrogen bonding interactions between carboxylic acid-functionalized worms (see Fig. S7†). The purpose of these experiments was to determine the critical gelation temperature (CGT), which is the temperature above which the dispersion is no longer a gel (*i.e.* where *G*′′ exceeds *G*′). A worm gel containing solely HOOC-PSMA_11_-PBzMA_65_ exhibited a CGT of 92 °C, whereas the CGT of H_3_COOC-PSMA_11_-PBzMA_65_ worm gel containing no carboxylic acid groups was significantly lower (41 °C). Similarly, a significant difference is observed for the critical gelation concentration (CGC), which is defined as the minimum concentration at which a free-standing gel can be obtained (see Fig. 3b and c). We have previously shown that reducing the copolymer concentration of worm gels leads to degelation due to the reduction in the number of inter-worm contacts that form the gel network.^75^ It is thus highly likely that reducing the copolymer concentration of the present worm dispersions would result in fewer inter-worm hydrogen bonds and hence weaker gels or macroscopic degelation. The CGC for the HOOC-PSMA_11_-PBzMA_65_ worm gel (*n* = 1.00) is 2–3% w/w, whereas that for the H_3_COOC-PSMA_11_-PBzMA_65_ worm gel (*n* = 0) is ∼10% w/w. For the former worm gel, the significantly lower CGC and higher CGT values observed are attributed to the additional hydrogen bonding interactions between adjacent HOOC-PSMA_11_-PBzMA_65_ worms, which act to reinforce the 3D gel network.^79^

**Fig. 4:**
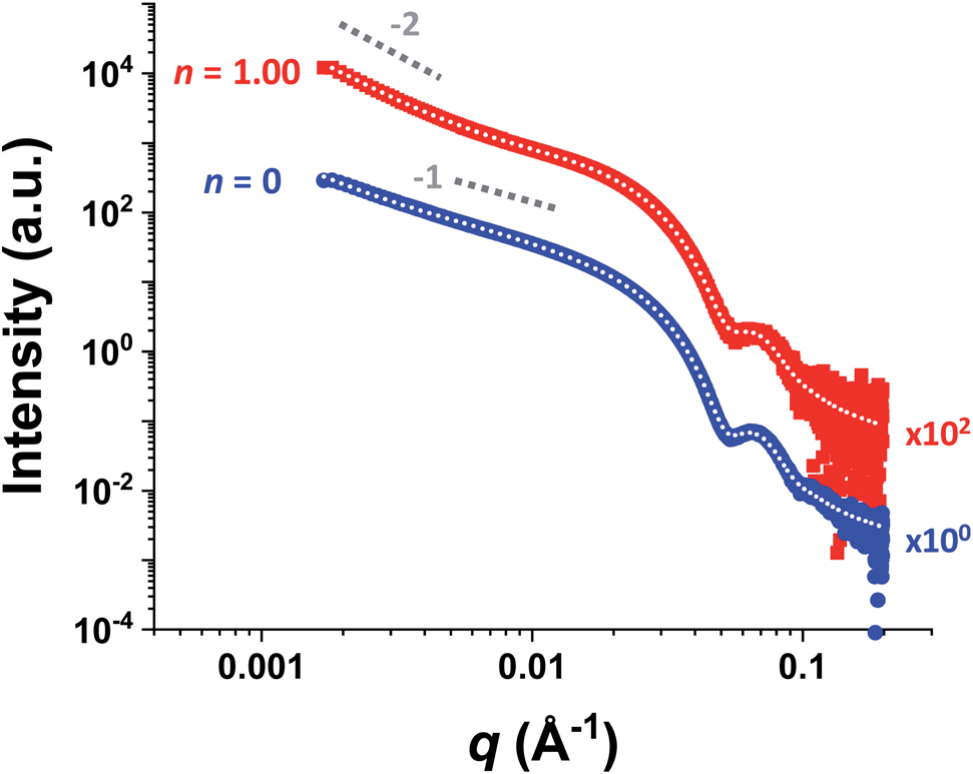
Small-angle X-ray scattering (SAXS) patterns recorded for 1.0% w/w dispersions of purely HOOC-PSMA_11_-PBzMA6_5_ worms (*n* = 1.00, red squares) and purely H_3_COOC-PSMA_11_-PBzMA_65_ worms (*n* = 0, blue circles) in *n*-dodecane. Indicative gradients of −1 and −2 are shown as a guide to the eye. Data fits using an established worm-like micelle model^78^ are shown as dotted white lines within the experimental data. A pronounced upturn at low *q* is observed for the upper pattern, indicating significantly stronger inter-worm interactions in this case. This is consistent with the postulated hydrogen-bonding interactions between neighboring worms (see Scheme 3). (N.B. the *n* = 1.00 pattern is offset by a factor of 10^2^ relative to the *n* = 0 pattern for the sake of clarity).

**Scheme 3:**
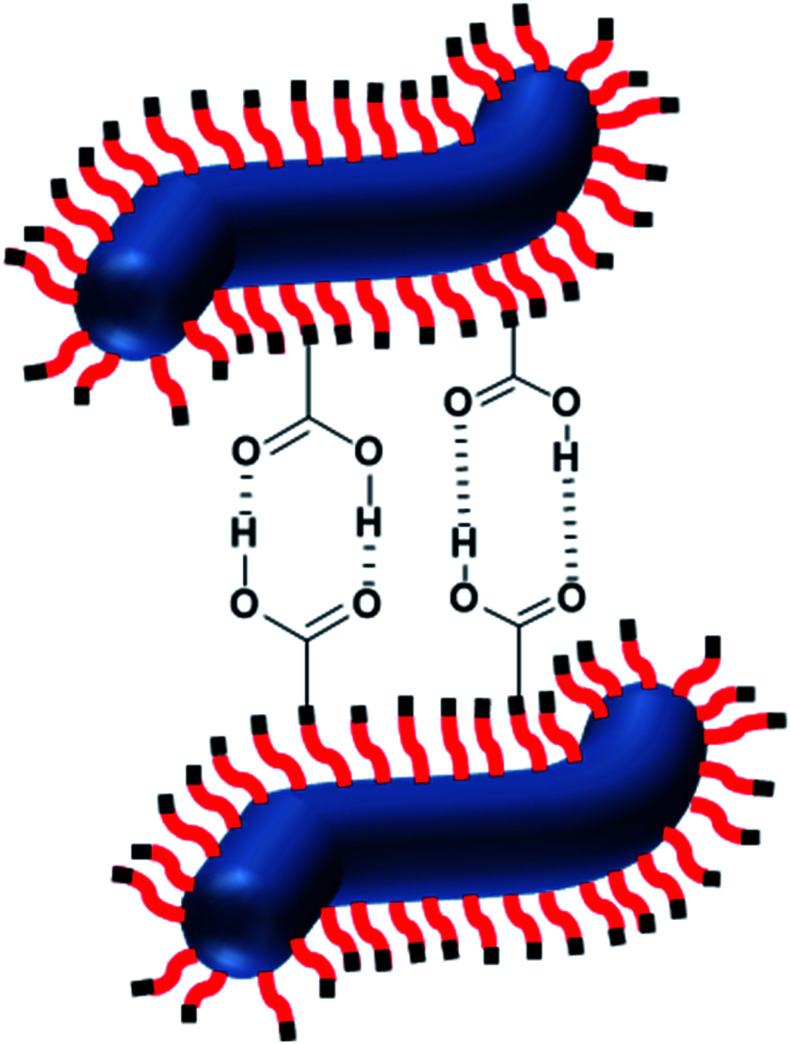
Schematic representation of the formation of hydrogen-bonded dimers between the carboxylic acid-functionalized described in this study.

For HOOC-PSMA_11_-PBzMA_65_ (*n* = 1.00) and H_3_COOC-PSMA_11_-PBzMA_65_ (*n* = 0) dispersions, SAXS patterns were recorded at a copolymer concentration of 1.0% w/w (see Fig. 4). In both cases, an approximate *I*(*q*) ∼ *q*^−1^ dependence was observed in the low *q* region, which is consistent with a well-defined worm-like morphology.^80^ Moreover, the local minima observed at high *q* indicate that these two types of worms exhibit the same mean worm core cross-sectional diameter. Indeed, fitting these data to a well-established worm-like micelle model^78^ confirmed that the mean overall worm thickness (*T*_worm_ = 2*R*_wc_ + 4*R*_g_, where *R*_wc_ is the mean worm core radius and *R*_g_ is the radius of gyration of the stabilizer chains) for these two samples were 16.8 and 16.6 nm, respectively. Furthermore, the mean worm length (*L*_worm_) was determined to be approximately 900 nm for both worms exhibit a pronounced upturn in X-ray scattering intensity dispersions. However, the HOOC-PSMA_11_-PBzMA_65_ (*n* = 1.00)at low *q* compared to the H_3_COOC-PSMA_11_-PBzMA_65_ (*n* = 0) worms. An *I*(*q*) = *Bq*^−*P*^ relationship was incorporated into the scattering model to account for this effect, whereby higher *P* values indicate a steeper slope in the low *q* region. Indeed, a *P* value of 1.95 was determined for the HOOC-PSMA_11_-PBzMA_65_ (*n* = 1.00) worms, whereas the H_3_COOC-PSMA_11_-PBzMA_65_ (*n* = 0) worms have a *P* value of only 1.63. This suggests significantly stronger inter-worm interactions for the former copolymer dispersion. More specifically, we hypothesize that such interactions involve the formation of hydrogen-bonded carboxylic acid dimers (see Scheme 3).

Recently we reported^75^ that, to a reasonable first approximation, the gelation behavior of block copolymer worms can be rationalized in terms of the percolation theory developed by Chatterjee for polydisperse rods.^81^ This suggests that multiple inter-worm contacts (rather than worm entanglements, which have been invoked to account for the gelation behavior observed for surfactant-based worms^82–84^) are sufficient to form a 3D gel network. Furthermore, the critical worm volume fraction, *φ*_c_, required for macroscopic gelation scales with the mean worm radius, *R*, and the weight-average worm length, *L*_w_ according to the simple relationship *φ*_c_ ∼ *R*/*L*_w_.^75^ Fitting the SAXS data shown in Fig. 4 to a well-established worm-like micelle model^78^ confirms that the mean dimensions (*i.e. R* and *L*_w_) for worms prepared using the carboxylic acid-based RAFT agent alone (*n* = 1.00) and the methyl ester-based RAFT agent alone (*n* = 0) are almost identical (see above). Thus, percolation theory predicts that these two types of worm gel should exhibit essentially the same behavior. However, the rheological data shown in Fig. 3a and digital images shown in Fig. 3b and c clearly indicate substantial differences in both the CGC (or *φ*_c_) and the CGT. This is consistent with additional attractive forces operating between neighboring worms, which leads to significantly stronger inter-worm interactions.

### Spectroscopic investigations of hydrogen bonding

We now present indirect spectroscopic evidence that such non-covalent interactions actually involve carboxylic acid dimer formation (see Scheme 3). Conventional hydrogen bonds such as those formed between water molecules exhibit enthalpies of dissociation of ∼23 kJ mol^−1^ at 298 K.^85^ However, the enthalpy of dissociation for carboxylic acid dimers is significantly stronger, typically ∼34 kJ mol^−1^ for acetic acid dimers in benzene at 298 K.^86^ In principle, the presence of hydrogen-bonded dimers could be confirmed by observing the splitting of the acid carbonyl stretching vibration into an IR-active and a Raman active component or by observing frequency shifts between the carboxylic acid-functionalized worms and the ester-functionalized worms. Unfortunately, obtaining direct spectroscopic evidence for the formation of carboxylic acid dimers between neighboring block copolymer worms in *n*-dodecane is not experimentally feasible for three reasons. First, there is only one carboxylic acid end-group per block copolymer chain. On the other hand, each chain contains on average 76 methacrylic ester repeat units, which absorb IR radiation at a very similar frequency (approximately 1700 cm^−1^ for carboxylic acids and 1720–1740 cm^−1^ for esters^87^). Second, unless the worms are aligned under flow, only a rather small proportion of carboxylic acid end-groups are expected to be involved in the formation of carboxylic acid dimers between neighboring worms when forming a percolating 3D gel network *via* inter-worms contacts.^75^ Finally, carboxylic acid dimer formation within individual worms is not spectroscopically distinguishable from that between neighboring worms, although only the latter species contributes to stronger worm gels.

Nevertheless, indirect evidence for carboxylic acid dimer formation can be obtained by FT-IR spectroscopy studies of each of the two PSMA_11_ precursors dissolved in *n*-dodecane (see Fig. S8†). In this case, there is one carboxylic acid end-group per eleven methacrylic ester repeat units, which makes it much easier to observe IR signatures arising from the former species. Thus, by working at a relatively high concentration (50% w/w), a wavenumber shift of approximately −10 cm^−1^ can be observed in the carbonyl region, which is in semi-quantitative agreement with theoretical calculations that predict a more pronounced red shift for carboxylic acid dimers compared to ester dimers (see details in the ESI†). Thus there is reasonable experimental and theoretical evidence to suggest that carboxylic acid-functionalized worms should exhibit much stronger hydrogen bonding interactions compared to ester-functionalized worms. This is expected to boost the bulk modulus observed for the former worm gels, as observed in the rheological studies reported herein.

## Conclusions

We report two complementary entropic mixing strategies for modulating the bulk modulus of sterically-stabilized poly(stearyl methacrylate)-poly(benzyl methacrylate) diblock copolymer worm gels *via* hydrogen bonding interactions. This is achieved simply by introducing carboxylic acid groups at the end of the poly(stearyl methacrylate) stabilizer chains, which leads to the formation of carboxylic acid dimers between neighboring worms in non-polar media. Unlike many other literature examples of hydrogen-bonded polymer systems, introducing this structural motif involves minimal synthetic effort because the RAFT polymerization chemistry employed to prepare these diblock copolymer worms typically utilizes carboxylic acid-based RAFT agents. Thus, using binary mixtures of carboxylic acid- and methyl ester-functionalized poly(stearyl methacrylate) stabilizers for the RAFT dispersion polymerization of benzyl methacrylate (Route 1) enables the storage gel modulus, *G*′, to be systematically varied from ∼4.5 kPa up to ∼114 kPa. A similar variation in gel strength can be achieved by exploiting the thermoreversible worm-to-sphere transition exhibited by such worm gels. Thus, mixing concentrated dispersions of free-flowing carboxylic acid- and methyl ester-functionalized spheres together at 110 °C leads to the formation of ‘hybrid’ segmented worms on cooling to ambient temperature *via* stochastic 1D fusion of multiple spheres (Route 2). Moreover, SAXS studies indicate that significantly stronger inter-worm interactions can be achieved when using a carboxylic acid-based RAFT agent for such worm gel syntheses, while rheological studies indicate both a significant reduction in critical gelation concentration (from approximately 10% w/w to 2–3% w/w) and a substantial increase in critical gelation temperature (from 41 °C to 92 °C). In summary, this study highlights how the introduction and judicious modulation of non-covalent interactions can be used to tune the physical properties of block copolymer worm gels, thus providing significant improvements in gel strength, gelation efficiency and gel cohesion.

## Data availability

There is no crystallographic data or software to deposit in a repository. Synthesis procedures are provided in detail in the Experimental Section found in the ESI.†

## Author contributions

M. J. D. and S. P. A. conceived the project. E. R. performed all syntheses and obtained NMR, GPC, rheology and FT-IR data. M. J. D. performed TEM and SAXS analyses. M. H. conducted quantum chemical calculations. All co-authors contributed to the writing, reviewing and editing of this manuscript.

## Conflicts of interest

There are no conflicts to declare.

## Supplementary Material

SC-012-D1SC03156B-s001
