# Supplementary material for: Tuning the properties of hydrogen-bonded block copolymer worm gels prepared via polymerization-induced self-assembly
Source: Chem Sci. 2021 Aug 5;12(36):12082–91. doi: 10.1039/d1sc03156b (PMC8457373; doi:10.1039/d1sc03156b)
Supplement: SC-012-D1SC03156B-s001 [file SC-012-D1SC03156B-s001.pdf]

Electronic Supplementary Material (ESI) for Chemical Science.
This journal is © The Royal Society of Chemistry 2021

Supporting Information for:

Tuning the Properties of Hydrogen-Bonded Block Copolymer Worm Gels Prepared via Polymerization-Induced Self-Assembly

Eleanor Raphael, Matthew J. Derry,* Michael Hippler, Steven P. Armes*

Experimental Section

Materials. Stearyl methacrylate (SMA) was purchased from Santa Cruz Biotechnology Ltd. (USA), *tert*-butyl peroxy-2-ethylhexanoate (T21s) initiator was obtained from AkzoNobel (The Netherlands) and 4-dimethylaminopyridine (DMAP) was acquired from Alfa Aesar (UK). THF and toluene were purchased from Fisher Scientific (UK), CD_2Cl_2 was purchased from Goss Scientific (UK) and a 4 cSt American Petroleum Institute (API) group III mineral oil was kindly provided by The Lubrizol Corporation Ltd (Hazelwood, Derbyshire, UK). All other materials were purchased from Sigma Aldrich (UK) and used as received, unless otherwise noted. Benzyl methacrylate (BzMA) was passed through basic alumina prior to use to remove its inhibitor. 4-Cyano-4-(2-phenylethanesulfanylthiocarbonyl)sulfanylpentanoic acid (PETTC) was synthesized according to the literature.[1]

Synthesis of HOOC-functionalized poly(stearyl methacrylate)$_{11}$ (HOOC-PSMA$_{11}$) macromolecular chain transfer agent (macro-CTA) via RAFT solution polymerization. SMA (40.10 g, 118 mmol), PETTC (5.03 g, 14.8 mmol), AIBN (0.486 g, 2.96 mmol; PETTC/AIBN molar ratio = 5.0) and toluene (52.0 mL) were weighed into a 250 mL round-bottomed flask, sealed and purged with nitrogen for 30 min while stirring. The degassed mixture was placed in an oil bath at 70 °C for 6 h before exposing to air (^{1}H NMR spectroscopy indicated an SMA monomer conversion of 76 %). The HOOC-PSMA product was precipitated into a ten-fold excess of ethanol twice in order to remove unreacted SMA monomer and then dried in a 30 °C vacuum oven overnight. The mean DP of the resulting HOOC-PSMA macro-CTA was 11, as judged by ^{1}H NMR spectroscopy. THF GPC analysis indicated an M_n of 5 400 gmol^{-1} and an M_w/M_n of 1.11.

Synthesis of H_3COOC-functionalized poly(stearyl methacrylate)$_{11}$ (H_3COOC-PSMA$_{11}$) macro-CTA. HOOC-PSMA$_{11}$ macro-CTA (10.0 g, 2.46 mmol), was weighed into a 250 mL round-bottomed flask, which was sealed and purged with nitrogen for 30 min. The minimum amount of anhydrous dichloromethane was added via a pre-degassed syringe to dissolve the polymer. Anhydrous methanol (0.215 mL, 5.31 mmol) and 4-dimethylaminopyridine (DMAP) (0.06 g, 0.491 mmol) were then added and the magnetically-stirred mixture was cooled to 0 °C. *N,N′*-Dicyclohexylcarbodiimide (DCC) (0.567 g, 2.75 mmol) was added over 5 min and the reaction flask was immersed in an ice bath overnight. The resulting solution was filtered to remove the insoluble by-product. Then the filtrate was precipitated into a ten-fold excess of ethanol (twice) to remain any remaining small molecule impurities before drying in a vacuum oven at 30°C for 16 h to afford the desired H_3COOC-PSMA$_{11}$ macro-CTA. ^{1}H NMR

spectroscopy analysis indicated a mean degree of esterification of 97% (see Figure S1) while THF GPC studies indicated an M_n of 5 500 g mol^{-1} and an M_w/M_n of 1.10.

Synthesis of HOOC-$PSMA_{11}$-$PBzMA_{65}$ diblock copolymer worms via RAFT dispersion polymerization of BzMA. The synthesis of HOOC-$PSMA_{11}$–$PBzMA_{65}$ diblock copolymer worms was conducted as follows: HOOC-$PSMA_{11}$ macro-CTA (0.26 g, 0.0640 mmol), BzMA (0.733 g, 4.16 mmol; target PBzMA DP = 65), T21s (307 μL of a 1.0 % v/v solution in *n*-dodecane, 0.0128 mmol; CTA/T21s molar ratio = 5.0) and additional *n*-dodecane (4.99 mL) were weighed into a 14 mL glass vial. This vial was sealed and the reaction mixture degassed with nitrogen for 30 min while stirring. The vial was then placed in an oil bath pre-set to 90 °C for 5 h. According to ^{1}H NMR spectroscopy studies, 97% BzMA conversion was achieved under such conditions. THF GPC analysis indicated an M_n of 15 100 g mol^{-1} and an M_w/M_n of 1.11 for the final HOOC-$PSMA_{11}$-$PBzMA_{65}$ worms.

Synthesis of H_3COOC-$PSMA_{11}$-$PBzMA_{65}$ diblock copolymer worms via RAFT dispersion polymerization of BzMA. The synthesis of H_3COOC-$PSMA_{11}$–$PBzMA_{65}$ diblock copolymer worms was conducted as follows: H_3COOC-$PSMA_{11}$ macro-CTA (0.26 g, 0.0638 mmol), BzMA (0.730 g, 4.14 mmol; target PBzMA DP = 65), T21s (306 µL of a 1.0 % v/v solution in n-dodecane, 0.0126 mmol; CTA/T21s molar ratio = 5.0) and additional n-dodecane (4.98 mL) were weighed into a 14 mL glass vial. This vial was sealed and the reaction mixture degassed with nitrogen for 30 min while stirring. The vial was then placed in an oil bath pre-set to 90 °C for 5 h. According to 1H NMR spectroscopy studies, 97% BzMA conversion was achieved under such conditions. THF GPC analysis indicated an Mn of 15 400 g mol^{-1} and an M_w/M_n of 1.13 for the final H_3COOC-$PSMA_{11}$-$PBzMA_{65}$ worms.

Synthesis of $PSMA_{11}$-$PBzMA_{65}$ diblock copolymer worms with binary mixtures of HOOC-$PSMA_{11}$ and H_3COOC-$PSMA_{11}$ macro-CTAs via RAFT dispersion polymerization of BzMA. The synthesis of $PSMA_{11}$–$PBzMA_{65}$ diblock copolymer worms using a 1:1 binary mixture of HOOC-$PSMA_{11}$ and H_3COOC-$PSMA_{11}$ macro-CTAs was conducted as follows: HOOC-$PSMA_{11}$ macro-CTA (0.130 g, 0.0320 mmol), H_3COOC-$PSMA_{11}$ macro-CTA (0.130 g, 0.0319 mmol; HOOC-$PSMA_{11}$/ H_3COOC-$PSMA_{11}$ molar ratio = 1.0), BzMA (0.731 g, 4.15 mmol; target PBzMA DP = 65), T21s (307 µL of a 1.0 % v/v solution in *n*-dodecane, 0.0128 mmol; CTA/T21s molar ratio = 5.0) and additional *n*-dodecane (4.98 mL) were weighed into a 14 mL glass vial. This vial was sealed and the reaction mixture degassed with nitrogen for 30 min with stirring. The vial was then placed in an oil bath pre-set to 90 °C. According to ^{1}H NMR spectroscopy studies, 97% BzMA monomer conversion was achieved within 5 h. THF GPC analysis indicated an M_n of 15 600 g mol^{-1} and an M_w/M_n of 1.11 for the final $PSMA_{11}$-$PBzMA_{65}$ worms. The same synthetic protocol was also used to prepare $PSMA_{11}$–$PBzMA_{65}$ diblock copolymer worms with either 3:1 or 1:3 binary mixtures of HOOC-$PSMA_{11}$ and H_3COOC-$PSMA_{11}$ macro-CTAs.

^{1}H NMR spectroscopy. All ^{1}H NMR spectra were recorded in either $CDCl_3$ or CD_2Cl_2 using a 400 MHz Bruker Avance-400 spectrometer with 64 scans being averaged per spectrum.

Gel permeation chromatography (GPC). GPC was used to assess the molecular weight distributions (MWDs) of the two $PSMA_{11}$ homopolymer precursors and the five diblock copolymers prepared using the binary mixture approach summarized in Scheme 1b. An Agilent 1260 Infinity system comprising an HPLC pump, two PL gel 5 µm (30 cm) Mixed C columns and a refractive index detector was run at 30 °C. The GPC eluent was HPLC-grade THF

containing 2.0% v/v triethylamine and 0.05 % w/v butylhydroxytoluene (BHT) at a flow rate of 1.0 mL min^{-1}. A series of twelve near-monodisperse poly(methyl methacrylate) standards with M_p values ranging from 625 to 2 480 000 g mol^{-1} were used for calibration. Varian Cirrus GPC software provided by the instrument manufacturer was used to analyze the chromatograms.

Transmission electron microscopy (TEM). TEM studies were conducted using a Philips CM 100 instrument operating at 100 kV and equipped with a Gatan 1 k CCD camera. A single droplet of each 0.10% w/w diblock copolymer dispersion was placed on a carbon-coated copper grid, allowed to dry and then exposed to ruthenium(VIII) oxide vapor for 7 min at 20 °C prior to analysis. This heavy metal compound acted as a positive stain for the core-forming PBzMA block so as improve electron contrast. The ruthenium(VIII) oxide was prepared as follows. Ruthenium(IV) oxide (0.30 g) was added to water (50 g) to form a black slurry; addition of sodium periodate (2.0 g) with stirring produced a yellow solution of ruthenium(VIII) oxide within 1 min.[2]

Small-angle X-ray scattering (SAXS). SAXS patterns were recorded at a synchrotron source (Diamond Light Source, station I22, Didcot, UK) using monochromatic X-ray radiation (X-ray wavelength λ = 0.999 Å, with scattering vector q ranging from 0.0018 to 0.15 Å^{-1}, where $q = 4\pi \sin \vartheta/\lambda$ and ϑ is one-half of the scattering angle) and a 2D Pilatus 2M pixel detector (Dectris, Switzerland). A glass capillary of 2 mm diameter was used as a sample holder and measurements were conducted on 1.0% w/w dispersions in *n*-dodecane. Scattering data were reduced and normalized with water being used for the absolute intensity calibration utilizing standard routines available at the beamline[3] and were further analyzed using Irena SAS macros for Igor Pro.[4]

Oscillatory rheology. All rheology measurements were recorded using a TA Instruments AR-G2 rheometer equipped with a 40 mm 2° aluminum cone and a variable temperature Peltier plate. For angular frequency sweeps, the storage (G') and loss (G'') moduli were measured from 0.1 to 100 rad s^{-1} at 20 °C using a fixed strain amplitude of 1.0 %. For temperature sweeps, G' and G'' were measured at a heating rate of 2 °C min^{-1}, and at an angular frequency of 10 rad s^{-1} and a fixed strain amplitude of 1.0%.

Fourier transform infrared (FT-IR) spectroscopy. All FT-IR spectra were recorded in absorbance mode using a Thermo Scientific Nicolet IS10 FT-IR spectrometer equipped with a Diamond ATR 'Golden Gate' accessory. A baseline was recorded before analyzing 50% w/w solutions of the HOOC-$PSMA_{11}$ and H_3COOC-$PSMA_{11}$ homopolymer precursors in *n*-dodecane at 20 °C. In each case, 1024 scans were averaged per spectrum. Omnic software provided by the instrument manufacturer was used to analyze the IR spectra.

Quantum-chemical calculations. High-level quantum chemical calculations have been performed using the General Atomic and Molecular Electronic Structure System (GAMESS) US program package, version 30 SEP 2018 (R3), on various 64 bit Windows and Linux personal computers.[5] The standard 6-31+G** basis set (Pople double split valence set with added polarization and diffuse functions) has been employed. Dynamic electron correlation has been included with 2nd order Møller-Plesset perturbation theory (MP2, frozen core). Geometry optimizations were performed with very tight convergence criteria. The nature of stationary states (minimum structures or transition states) was confirmed by frequency calculations with numerical derivatives of the gradients, where positive and negative Cartesian displacements were enforced to increase the accuracy. A

vibrational scaling factor of 0.95 was used to partially compensate for anharmonicities and other effects for zero-point energies and thermodynamic properties. Thermodynamic properties refer to 1 atm and 298.15 K using the usual ideal gas, rigid rotor and harmonic normal mode approximations in statistical thermodynamics. No attempts were made to include solvent effects in these calculations. Calculations on molecular clusters are affected by the basis set superposition error (BSSE). For energy, geometry optimization (gradients) and vibrational (Hessian) calculations on molecular clusters, the BSSE was corrected using a suite of programmes,[6-9] implementing the counterpoise correction procedure (CP) as proposed by Boys and Bernardi[10] within GAMESS.

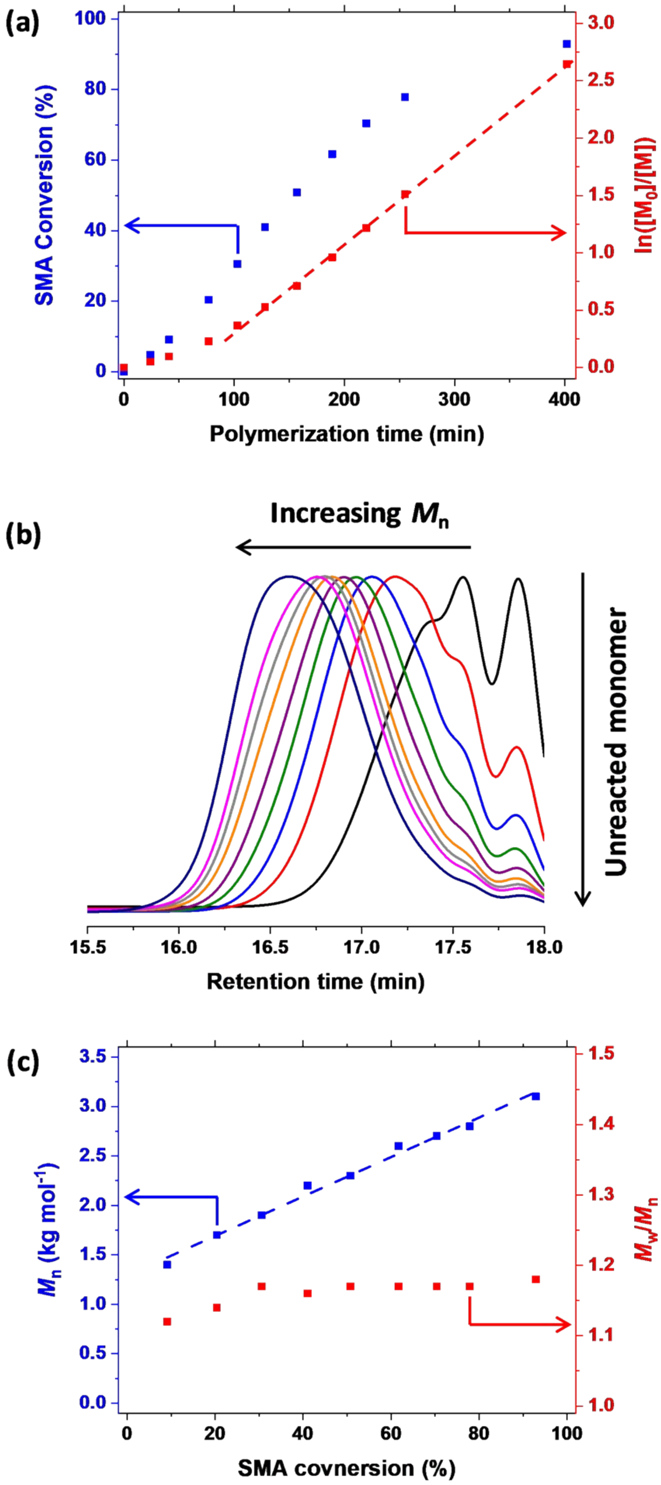

Figure S1. Kinetic study of the RAFT solution polymerization of stearyl methacrylate (SMA) using 4-cyano-4-(2-phenylethanesulfanylthiocarbonyl)sulfanylpentanoic acid (PETTC) in toluene at 50% w/w and 70 °C: (a) conversion vs. time curve and corresponding semi-logarithmic plot, (b) THF GPC curves and (c) evolution in M_n and M_w/M_n with monomer conversion.

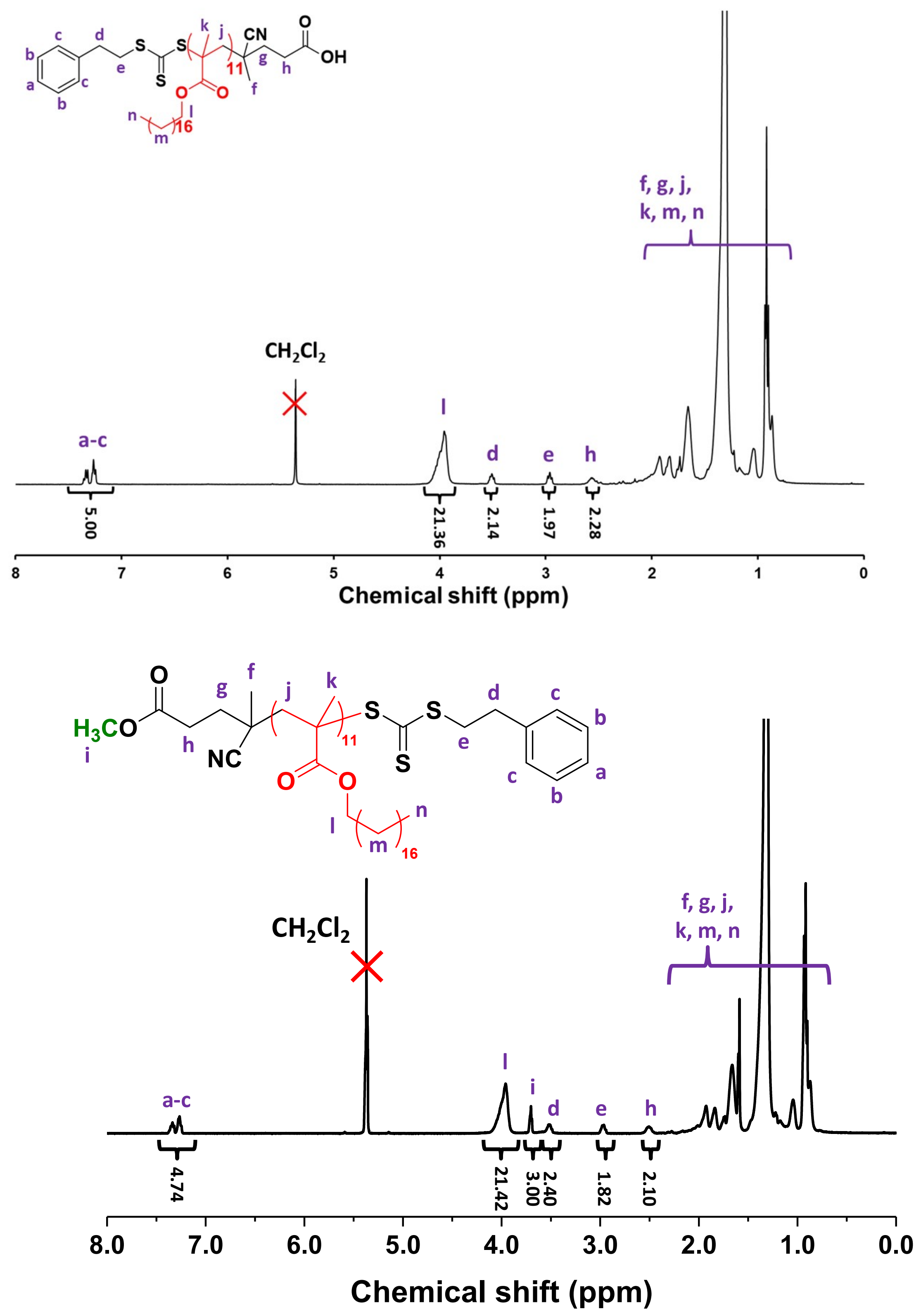

Figure S2. Assigned ^{1}H NMR spectrum (CD_2Cl_2) for the (top) HOOC-$PSMA_{11}$ macro-CTA and (bottom) **H_3C**OOC-$PSMA_{11}$ macro-CTA.

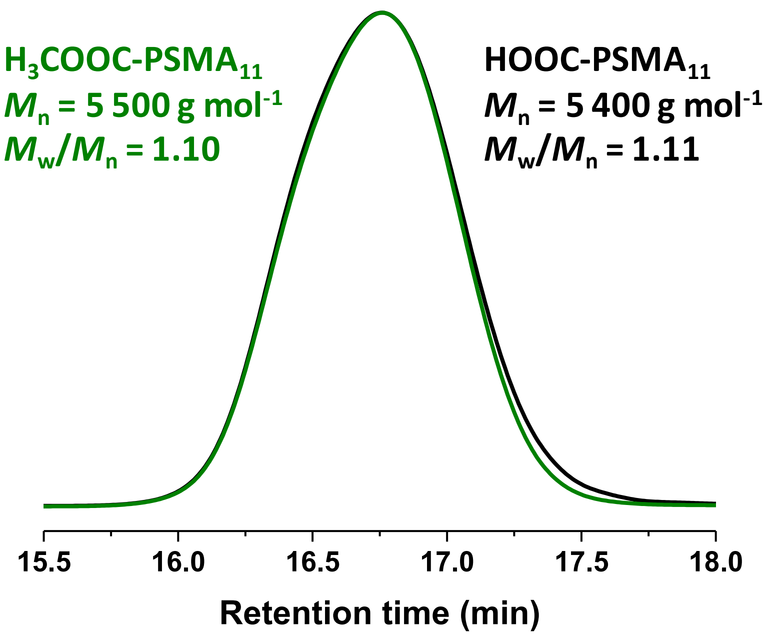

Figure S3. THF GPC curves recorded for HOOC-PSMA$_{11}$ and **H$_3$C**OOC-PSMA$_{11}$ macro-CTAs.

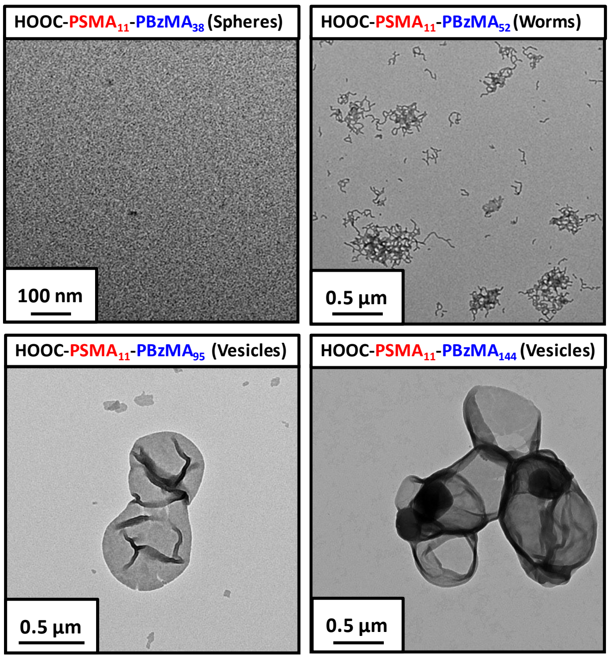

Figure S4. Representative transmission electron micrographs recorded for various HOOC-PSMA$_{11}$-PBzMA$_x$ diblock copolymer nano-objects (x = 38, 52, 95 or 144) synthesized via RAFT dispersion polymerization of BzMA in *n*-dodecane at 90 °C. Conditions: 20% w/w, [HOOC-PSMA$_{11}$]/[T21s] molar ratio = 5.0, reaction time = 5 h.

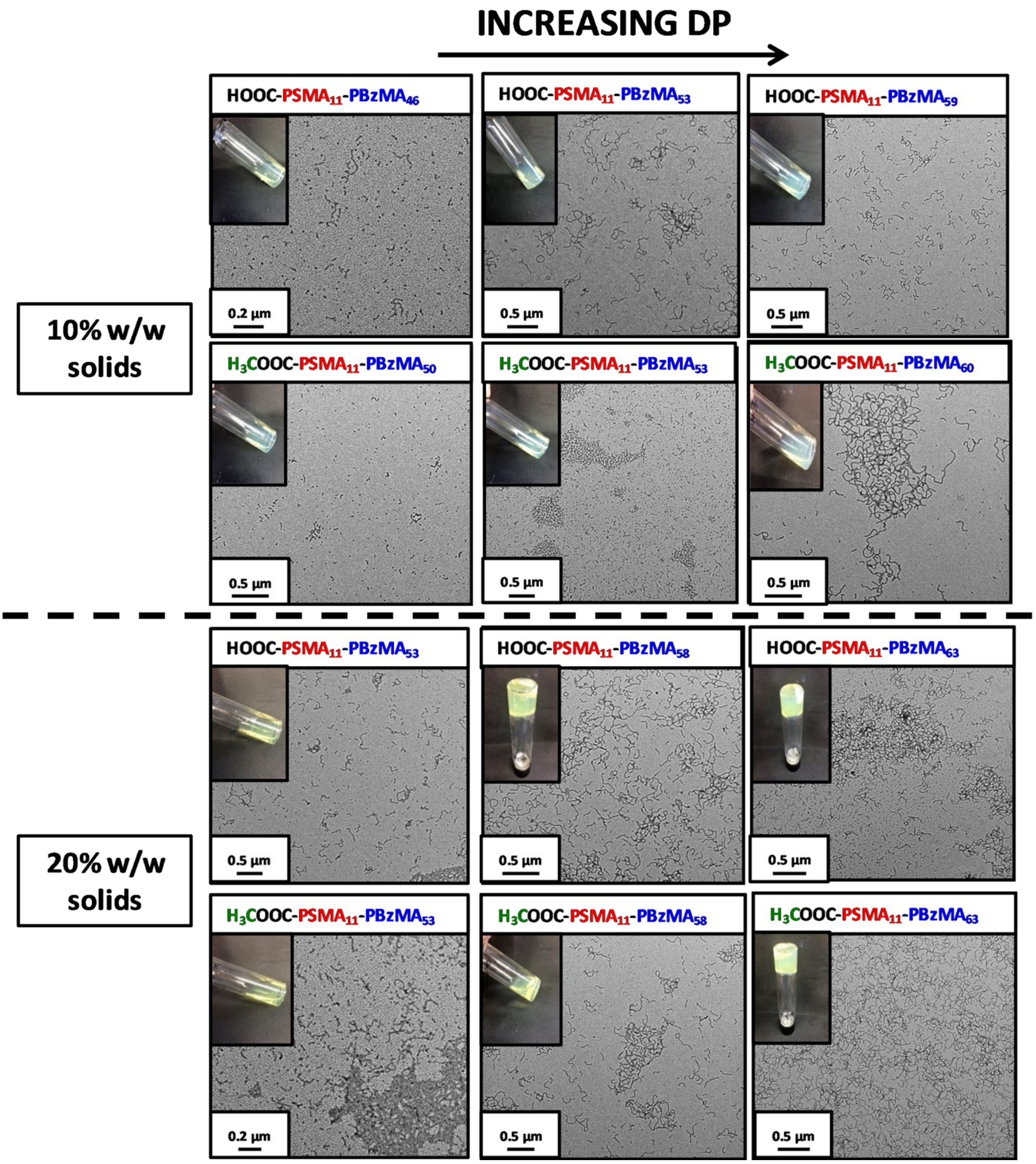

Figure S5. Transmission electron micrographs recorded for various HOOC-PSMA$_{11}$-PBzMA$_x$ and H$_3$COOC-PSMA$_{11}$-PBzMA$_x$ diblock copolymer nano-objects prepared via RAFT dispersion polymerization of BzMA in *n*-dodecane at either 10% w/w (top) or 20% w/w (bottom). Conditions: [PSMA$_{11}$]/[T21s] molar ratio = 5.0, 90 °C, 5 h.

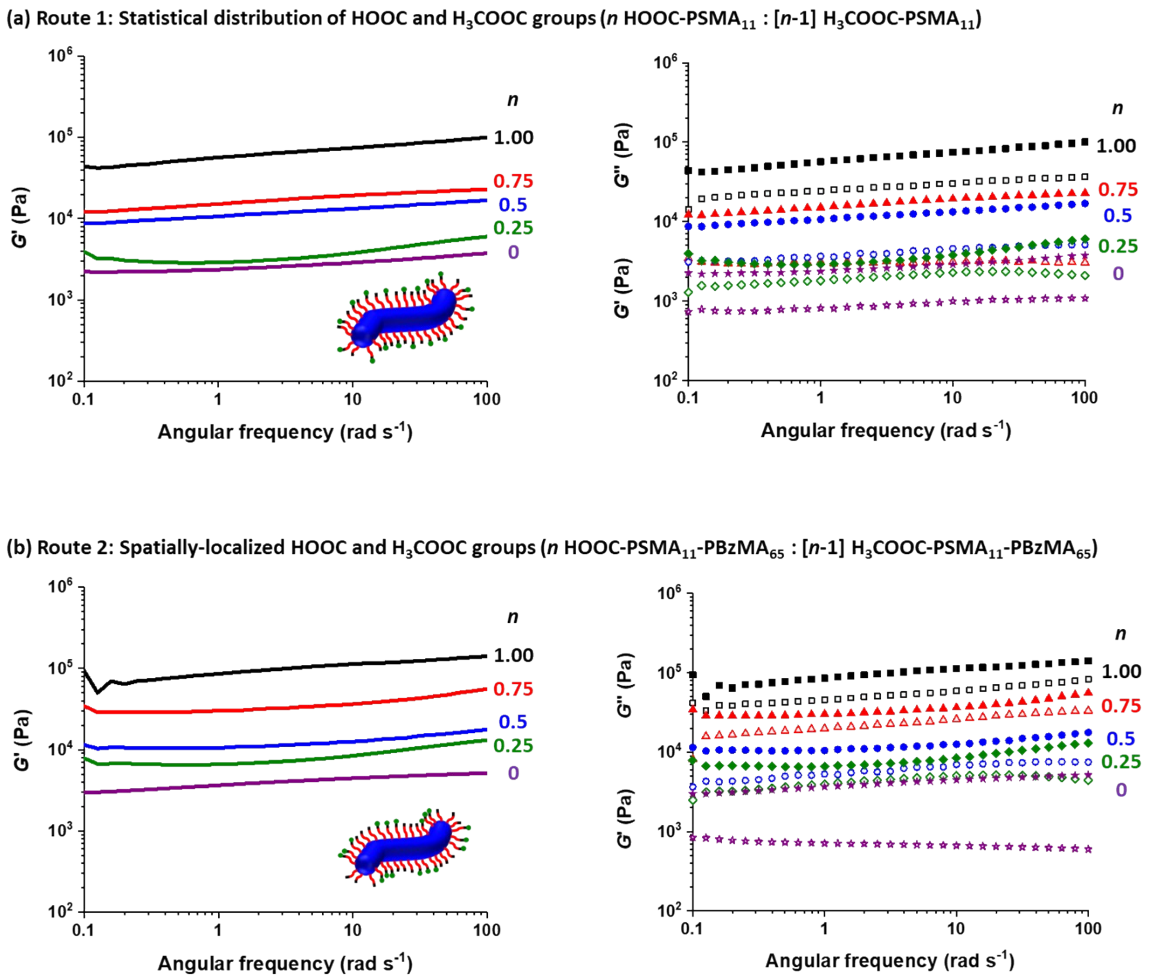

Figure S6. Frequency dependence of (left) the storage modulus, *G*′, and (right) both *G*′ (filled symbols) and the loss modulus, *G*″ (open symbols), obtained for a series of five $PSMA_{11}$-$PBzMA_{65}$ worm gels prepared with varying proportions of carboxylic acid end-groups by (a) **Route 1** (statistical distribution of stabilizer chains, black data set) and (b) **Route 2** (spatially-localized patches of HOOC-functionalized stabilizer chains on 'hybrid' segmented worms, red data set).

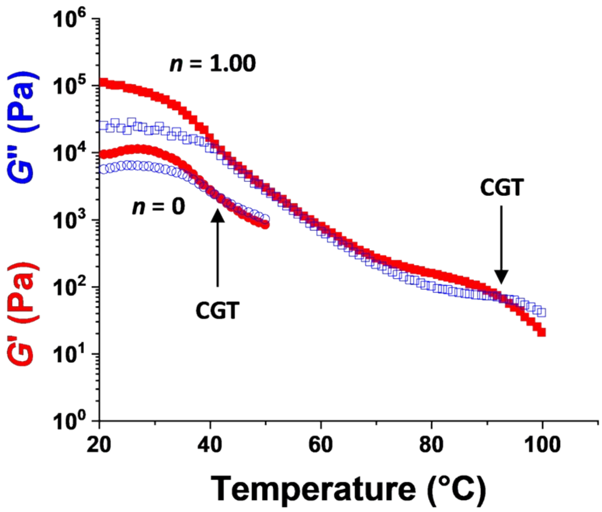

Figure S7. Temperature dependence for both storage (*G*’, red solid symbols) and loss (*G*”, blue open symbols) moduli observed for 20% w/w dispersions comprising solely HOOC-$PSMA_{11}$-$PBzMA_{65}$ (n = 1.00, squares) and solely H_3COOC-$PSMA_{11}$-$PBzMA_{65}$ (n = 0, circles) diblock copolymer nano-objects at a heating rate of 2 °C min^{-1}, an angular frequency of 10 rad s^{-1} and a fixed strain amplitude of 1.0%.

Spectroscopic investigations of hydrogen bonding

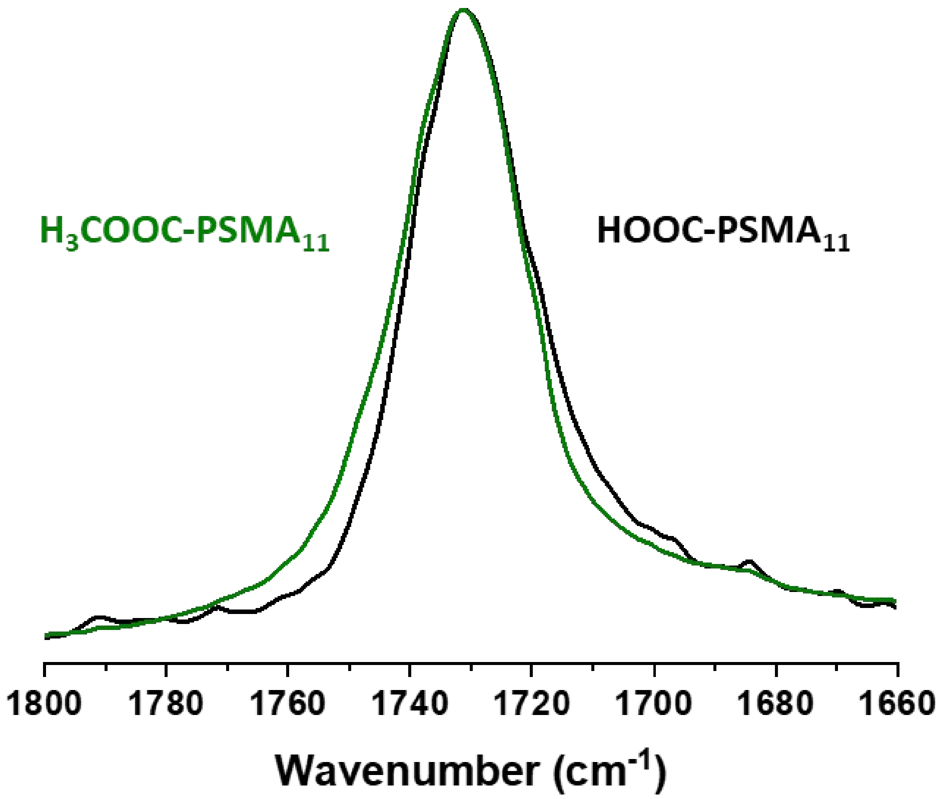

Figure S8. FT-IR spectra recorded at 298 K for 50% w/w solutions of HOOC-PSMA$_{11}$ (black trace) and H$_3$COOC-PSMA$_{11}$ (green trace) homopolymer precursors in *n*-dodecane.

In order to support the hypothesis of the formation of carboxylic acid dimers and also to guide spectroscopic measurements, we performed high-level quantum chemical calculations (MP2/6-31G(d,p,)++, CP-corrected) on two model systems. In these calculations, acetic acid is used as a proxy for the carboxylic acid-functionalized polymer chains and methyl acetate is employed as a model for the methyl-ester-capped polymer chains.

As expected, the most stable conformation for acetic acid is characterized by strong hydrogen-bonded dimers (see Scheme 3 in the main manuscript). Given its C_i inversion symmetry, the vibrational exclusion rule applies to this structure, thus vibrations are either IR-active (anti-symmetric) or Raman-active (symmetric). The binding energy, ΔE_{el} (essentially the dissociation energy at 0 K) is calculated to be –56.2 kJ mol^{-1} (and ΔE_0 = –50.2 kJ mol^{-1} including the vibrational zero-point energy). These results are consistent with prior theoretical and experimental studies on acetic acid dimers. For example, B3LYP/6-31G** calculations by Chocholoušová and co-workers suggest ΔE_{el} = –66.3 kJ mol^{-1}, although this is a somewhat lower level theory.[11] Similarly, an experimental study of acetic acid dimers in the gas phase by Jaffe and Rose indicated ΔH^{o} (298 K) = –60.3 kJ mol^{-1}.[12] The ester carbonyl band is split into an IR-active anti-symmetric stretch at 1787.5 cm^{-1} and an IR-inactive (but Raman-

active) symmetric stretch at 1753.9 cm^{-1}. The wavenumber for the IR-active component is red-shifted by –29.9 cm^{-1} compared to the monomer. However, this shift is expected to be smaller at 298 K owing to hot bands.[9]

The most stable equilibrium configuration of the methyl acetate dimer also involves hydrogen-bonded dimers (see Figure S9). However, hydrogen bonds between C=O and H-C are much weaker than those involving O-H or N-H groups.[6-9] The calculated binding energy is ΔE_{el} = –12.5 kJ mol^{-1}, and ΔE_0 = –11.1 kJ mol^{-1} (including vibrational zero-point energies). To a first approximation, the ester carbonyl stretching vibration is split into an IR-active antisymmetric stretching at 1789.0 cm^{-1}, and an IR-inactive (but Raman-active) symmetric stretching at 1782.0 cm^{-1}. The wavenumber shift of the IR-active component compared to the monomer is only –5.0 cm^{-1}, which reflects the much weaker hydrogen-bonding in this case. In practice, this shift will be even smaller at 298 K owing to hot bands.[9] The Raman spectrum should exhibit a more pronounced shift of –12.0 cm^{-1} (but again this shift is expected to be smaller at 298 K owing to hot bands).

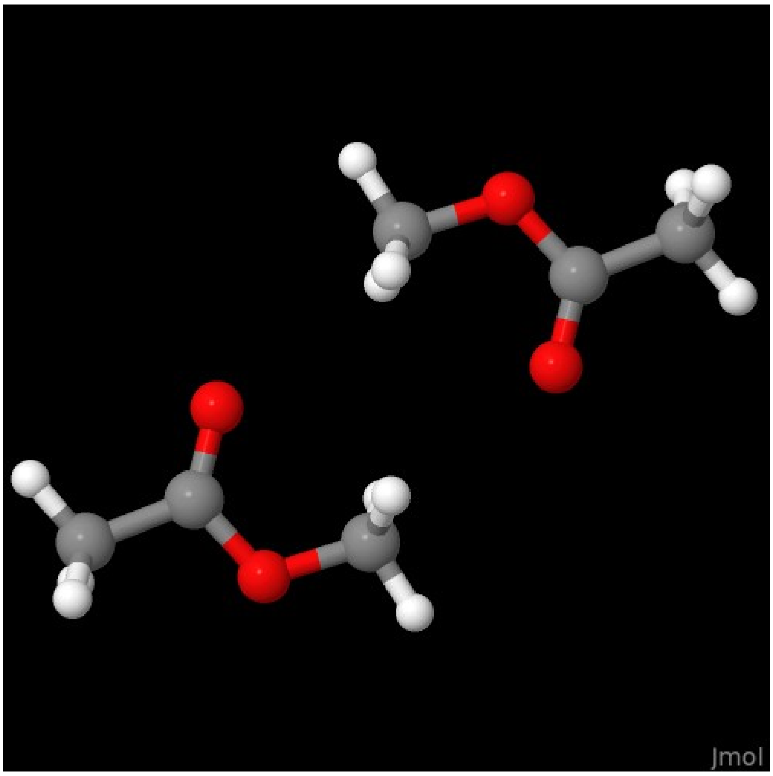

Figure S9. Calculated configuration of cyclic methyl acetate dimer, MP2/6-31G(d,p,)++, CP-corrected.

Worm-like micelle SAXS model

Programming tools within the Irena SAS Igor Pro macros[3] were used to implement the scattering models.

In general, the intensity of X-rays scattered by a dispersion of nano-objects [as represented by the scattering cross-section per unit sample volume, $\frac{d\Sigma}{d\Omega}(q)$] can be expressed as:

$$\frac{d\Sigma}{d\Omega}(q) = NS(q)\int_0^\infty ...\int_0^\infty F(q,r_1,...,r_k)^2\Psi(r_1,...,r_k)dr_1,...,dr_k \qquad S1$$

where $F(q,r_1,...,r_k)$ is the form factor, $r_1,...,r_k$ is a set of *k* parameters describing the structural morphology, $\Psi(r_1,...,r_k)$ is the distribution function, *S*(*q*) is the structure factor and *N* is the number density of nano-objects per unit volume expressed as:

$$N = \frac{\varphi}{\int_0^\infty ...\int_0^\infty V(r_1,...,r_k)\Psi(r_1,...,r_k)dr_1,...,dr_k} \qquad S2$$

where $V(r_1,...,r_k)$ is the volume of the nano-object and φ is its volume fraction within the dispersion. It is assumed that *S*(*q*) = 1 at the sufficiently low copolymer concentrations used in this study (1.0% w/w).

The worm-like micelle form factor for Equation S1 is given by:

$$F_{w\_mic}(q) = N_w^2\beta_s^2F_{sw}(q) + N_w\beta_c^2F_c(q,R_g) + N_w(N_w - 1)\beta_c^2S_{cc}(q) + 2N_w^2\beta_s\beta_cS_{sc}(q) \qquad S3$$

where R_g is the radius of gyration of the coronal steric stabilizer block (in this case, $PSMA_{11}$). The X-ray scattering length contrasts for the core and corona blocks are given by $\beta_s = V_s(\xi_s - \xi_{sol})$ and $\beta_c = V_c(\xi_c - \xi_{sol})$ respectively. Here, ξ_s, ξ_c and ξ_{sol} are the X-ray scattering length densities of the core block (ξ_{PBzMA} = 10.38 x 10^{10} cm^{-2}), corona block (ξ_{PSMA} = 9.24 x 10^{10} cm^{-2}) and *n*-dodecane solvent (ξ_{sol} = 7.32 x 10^{10} cm^{-2}), respectively. V_s and V_c are the volumes of the core block (V_{PBzMA}) and the corona block (V_{PSMA}), respectively. The self-correlation term for the worm core cross-sectional volume-average radius R_{sw} is:

$$F_{sw}(q) = F_{worm}(q,L_w,b_w)A_{CSworm}^2(q,R_{sw}) \qquad S4$$

where

$$A_{CSworm}^2(q,R_{sw}) = \left[2\frac{J_1(qR_{sw})}{qR_{sw}}\right]^2 \qquad S5$$

and J_1 is the first-order Bessel function of the first kind, and a form factor $F_{worm}(q,L_w,b_w)$ for self-avoiding semi-flexible chains represents the worm-like micelles, where b_w is the Kuhn length and L_w is the mean contour length. A complete expression for the chain form factor can be found elsewhere.[6]

The mean aggregation number of the worm-like micelle, N_w, is given by:

$$N_w = (1 - x_{sol})\frac{\pi {R_{sw}}^2 L_w}{V_s} \qquad S6$$

where x_{sol} is the volume fraction of solvent within the worm-like micelle cores, which was found to be zero in all cases. The possible presence of semi-spherical caps at both ends of each worm is neglected in this form factor.

A polydispersity for one parameter (R_{sw}) is assumed for the micelle model, which is described by a Gaussian distribution. Thus, the polydispersity function in Equation S1 can be represented as:

$$\Psi(r_1) = \frac{1}{\sqrt{2\pi {\sigma_{R_{sw}}}^2}} exp\left(- \frac{(r_1 - R_{sw})^2}{2{\sigma_{R_{sw}}}^2} \right) \qquad S7$$

where $\sigma_{R_{sw}}$ is the standard deviation for R_{sw}. In accordance with Equation S2, the number density per unit volume for the worm-like micelle model is expressed as:

$$N = \frac{\varphi}{\int_0^\infty V(r_1)\Psi(r_1)dr_1} \qquad S8$$

where φ is the total volume fraction of copolymer in the worm-like micelles and $V(r_1)$ is the total volume of copolymer in a worm-like micelle $[V(r_1) = (V_s + V_c)N_w(r_1)]$.

References

1. M. J. Rymaruk, K. L. Thompson, M. J. Derry, N. J. Warren, L. P. D. Ratcliffe, C. N. Williams, S. L. Brown and S. P. Armes, *Nanoscale*, 2016, **8**, 14497-14506.
2. J. S. Trent, *Macromolecules*, 1984, **17**, 2930-2931.
3. B. R. Pauw, A. J. Smith, T. Snow, N. J. Terrill and A. F. Thunemann, *Journal of Applied Crystallography*, 2017, **50**, 1800-1811.
4. J. Ilavsky and P. R. Jemian, *Journal of Applied Crystallography*, 2009, **42**, 347-353.
5. M. W. Schmidt, K. K. Baldridge, J. A. Boatz, S. T. Elbert, M. S. Gordon, J. H. Jensen, S. Koseki, N. Matsunaga, K. A. Nguyen, S. Su, T. L. Windus, M. Dupuis and J. A. Montgomery Jr, *Journal of Computational Chemistry*, 1993, **14**, 1347-1363.
6. M. Hippler, *The Journal of Chemical Physics*, 2005, **123**, 204311.

7. S. Chung and M. Hippler, *The Journal of Chemical Physics*, 2006, **124**, 214316.
8. M. Hippler, *The Journal of Chemical Physics*, 2007, **127**, 084306.
9. M. Hippler, S. Hesse and M. A. Suhm, *Physical Chemistry Chemical Physics*, 2010, **12**, 13555-13565.
10. S. F. Boys and F. Bernardi, *Molecular Physics*, 1970, **19**, 553-566.
11. J. Chocholoušová, J. Vacek and P. Hobza, *The Journal of Physical Chemistry A*, 2003, **107**, 3086-3092.
12. D. A. Jaffe and N. J. Rose, *Spectrochimica Acta Part A: Molecular Spectroscopy*, 1991, **47**, 1695-1705.
